# Nematicidal Potential of Green Silver Nanoparticles Synthesized Using Aqueous Root Extract of *Glycyrrhiza glabra*

**DOI:** 10.3390/nano12172966

**Published:** 2022-08-27

**Authors:** Kanika Rani, Nisha Devi, Prakash Banakar, Pushpa Kharb, Prashant Kaushik

**Affiliations:** 1 Department of Molecular Biology, Biotechnology and Bioinformatics, Chaudhary Charan Singh Haryana Agricultural University, Hisar 125004, India; kvats54@gmail.com (K.R.); nishu.sharma57605@gmail.com (N.D.); 2 Department of Nematology, Chaudhary Charan Singh Haryana Agricultural University, Hisar 125004, India; prakashbanakar@gmail.com; 3 Kikugawa Research Station, Yokohama Ueki, Kikugawa 439-0031, Japan

**Keywords:** *Meloidogyne incognita*, *Glycyrrhiza glabra*, silver nanoparticles, nematicidal potential, nanotoxicity, green synthesis, RT-PCR

## Abstract

*Meloidogyne incognita* (root-knot nematode) is a devastating soil-borne pathogen which can infect almost all cultivated plants around the globe, expediting huge pecuniary losses. The purpose of current study was to use the aqueous root extract of *Glycyrrhiza glabra* for synthesizing silver nanoparticles (GRAgNPs) and assess their nematicidal potential against *M. incognita* by in vitro methods, including hatching inhibition and mortality assays. The active uptake of FITC labeled GRAgNPs by the nematode and their effect on the expression of selected genes involved in oxidative stress and DNA damage repair were also studied. An HRTEM micrograph confirmed their spherical morphology with sizes ranging from 9.61 nm to 34.735 nm. Complete inhibition of egg-hatching was observed after 48 h of treatment with as low as 10.0 ppm of GRAgNPs. In addition, 100% mortality was recorded at the lowest dose of 6.0 ppm, after 12 h of treatment. The LC-50 for GRAgNPs was found to be 0.805 ± 0.177 ppm at *p* < 0.0001, R^2^ = 0.9930, and α = 0.05. The expression of targeted genes (*skn-1*, *mev-1*, *sod-3*, *dhs-23*, *cyp-450*, *xpa*, *cpr-1*, *gst-n*, and *ugt*) was significantly enhanced (1.09–2.79 folds), at 1.0 ppm (α = 0.05, 95% CI) GRAgNPs treatment. In conclusion, GRAgNPs performed efficaciously and considerably in contrast to chemical nematicide and commercial silver nanoparticles (CAgNPs) and might be used as a promising alternative as relatively lower concentration and short exposure time were enough to cause higher mortality and nanotoxicity in nematodes.

## 1. Introduction

Nanotechnology is emerging as a fast-growing field with a wide range of applications in various science and technology domains, including medicine, pharmaceuticals, engineering, agriculture, food industry, electronics, etc. [1,2]. Agricultural production needs to be enhanced to meet the demand of the ever-increasing world population. This could be achieved with the immense potential of nanotechnology in various aspects of agriculture such as the management of pests, pathogens, weeds and diseases, plant protection, maintenance of soil and water health, pollution monitoring, improving quality of crops, and developing nano-sensors [3,4]. Silver nanoparticles (AgNPs) have been reported to exhibit peculiar properties with a wide range of applications in agriculture, water purification, air filtration, pharmaceutics, catalysis, and textile industries [5,6] and thereby provide useful platform to explore various interdisciplinary fields. Further, AgNPs can be used as effective antibacterial, antifungal, insecticidal and nematicidal agents [4,7].

There are several physical, chemical and biological methods for the synthesis of AgNPs [6]. The physical methods include evaporation-condensation, laser ablation, ball-milling, spray pyrolysis, sputtering etc., whereas chemical methods include chemical vapor deposition, wet chemical synthesis, sol-gel method, reverse micelle etc. However, shortcomings such as the requirement of high energy, pressure, complex and expensive instrumental designing, and poor yield are associated with physical methods [6], while the use of toxic, volatile and hazardous chemical reagents and formation of non-eco-friendly by-products are major drawbacks in chemical methods [8]. Biological synthesis methods include usage of entities such as bacteria, algae, fungi, and plants. Microbe-mediated synthesis has limitations such as cost of isolation of microbes, tedious maintenance of cell cultures, high aseptic conditions, and biohazard etc. [8] Green synthesis with the usage of plant extract is an eco-friendly, cost-effective and time saving alternative approach without using toxic chemicals [6,9] and thus, potentially advantageous over the other methodologies.

Plant-parasitic nematodes (PPNs) are considered as a serious threat to agriculture as these soil borne pathogens cause root infection in the majority of crops affecting their yield and quality. Among these, the root-knot nematode (RKN) belonging to genus, *Meloidogyne,* can infect almost all cultivated plants worldwide [10] and *M. incognita* is the most devastating species in this genus. The eggs of RKN rapidly develop to first stage juvenile (J1s), which molt to motile second stage juveniles (J2s) and start the plant infection. J2s invade roots, migrate to vascular tissues and form ‘root knots’ or ‘galls’ as well as develop special ‘giant feeding cells’, permanently affecting the nutrient and water uptake in their host [11]. Speedy population growth of *M. incognita* due to high female fecundity and completion of two or more life cycles in a single growing season, leads to declining crop yield and causing huge financial adversity to farmers worldwide [12]. The use of chemical based synthetic nematicides is still the primary managing technique against PPNs. However, these nematicides are expensive, toxic, and hazardous in nature and raise concerns for residual toxicity, environmental pollution, and associated health risks [13]. Therefore, alternative eco-friendly approaches for pest management are required.

Green synthesized AgNPs could be an efficient solution for this problem. Phyto-constituents used for the green synthesis can act as natural reducing and capping agents and help in the production of stable NPs [14]. *Urtica urens* based AgNPs have been reported to be eleven-fold more toxic to eggs of *M. incognita* in comparison to plant extracts alone [15]. Similarly, *Conya dioscoridis* based AgNPs exhibited a similar nematicidal effect on reference Rugby [16]. Nematotoxicity could be due to induced oxidative stress generated by AgNPs [8,17] and was recorded in in-vitro and pot experiments.

*Glycyrrhiza glabra,* commonly known as Mulhathi, is an important traditional medicinal plant worldwide with high ethnopharmacological value. Its dried roots have been used by Chinese, Indian, Egyptian, Roman and Greek civilizations for the treatment of throat and bronchial infections for many decades [14]. Roots of *G. glabra* contain flavonoids (liquiritin, liquiritigenin, glabridin), triterpenoids (glycyrrhizin), phenolics (glycybridins), saponin (glycyrrhizic acid, glycyrrhetinic acid), coumarins, alkaloids, antioxidants, glycosides, tannins, sterols and steroids [18,19]. There are several reports on anti-microbial and anti-viral efficacy of the aqueous root extract of *G. glabra*, but there is not even a single report on its nematicidal potential. The present study was aimed to use the aqueous root extract of *G. glabra* for synthesizing AgNPs (GRAgNPs) and evaluate their bio-efficacy against root-knot nematode, *M. incognita* by in vitro methods. We also investigated the potential effect of these GRAgNPs on the expression of selected genes involved in oxidative stress and DNA damage repair. To the best of our knowledge, it is the first report on this plant in the context of the nematicidal potential of GRAgNPs against *M. incognita* and their impact on the expression of selected genes involved in oxidative stress and DNA damage repair.

## 2. Materials and Methods

### 2.1. Materials

Roots of *G. glabra* (var. HM-1) were collected from the research farm of CCS HAU, Hisar, India. Commercial nematicide, Velum prime (VP) was obtained from Department of Nematology, CCS HAU, Hisar. Silver nitrate (AgNO_3_, analytical grade) and commercial silver nanoparticles (CAgNPs) were obtained from Sigma-Aldrich Chemical Co. (St. Louis, MO, USA). As per product specification, the purchased CAgNPs were <100 nm in size and almost spherical in shape (TEM analysis) with λ_max_ of 422 nm (UV-VIS analysis) and were coated with polyvinylpyrrolidone (PVP).

### 2.2. Synthesis and Characterization of GRAgNPs

The roots collected from the field were thoroughly washed with running tap water followed by distilled water and kept in an oven at 60 °C for 70–72 h for drying. The dried root material was grinded using a grinding mill (FOSS, Cyclotech^TM^ 1093) and stored in a wide mouth bottle (Medical Grade, HDPE). In addition, 5% *w*/*v* aqueous root extract (RE) was prepared by boiling and the extract was filtered through 0.22 µ filter and stored at 4 °C for further use. For the synthesis of GRAgNPs, 10 mL of aqueous root extract was added to 100 mL of 1 mM AgNO_3_ and kept in an orbital shaker at 500 rpm for 1 h at room temperature. The mixture was then incubated at 80 °C for 12 h.

The absorption spectra of RE, GRAgNPs, and AgNO_3_ (used as blank) were recorded with a UV-VIS spectrophotometer (UV-2600, Shimadzu Corporation, Tokyo, Japan) at 350–500 nm wavelength with the threshold of 0.001 and accumulation time 0.1 s. The particle size distribution, polydispersity index (PDI) and zeta potential of GRAgNPs were determined by a Nanoparticle Size Analyzer (Microtrac Nanotrac Wave II, Verder Scientific Pvt. Ltd., Hyderabad, India) at a field strength of 10 kV. Average hydrodynamic size was determined by utilizing the freshly synthesized sample without any further dilution and sonication. The colloidal monodispersive nature of GRAgNPs was confirmed by polydispersity index (PDI). Fourier transform infrared (FTIR) spectrometer (Thermo Scientific Nicolet iS50 FT-IR, Thermo Fisher Scientific, St. Bend, OR, USA) was used to investigate the chemical functional groups of root powder of *G. glabra* var. HM-1 involved in reduction and capping of GRAgNPs in the region of 4000–400 cm^−1^ using KBr pellet. The topographic detailing of surface of GRAgNPs was undertaken using a field emission scanning electron microscope, FE-SEM (FEI Nova NanoSEM 450, FEI company, Thermo Fisher Scientific, St. Bend, OR, USA) and measurements were carried out by Au-coating of NPs at an accelerating voltage of 15 kV in a dwell time of 10 µs in an immersed lens mode using a TLD detector. The elemental composition and its abundance were measured by Energy Dispersive X-ray (EDX) detector, QUANTAX (Bruker Corporation, Billerica, MA, USA) attached to SEM. EDX analysis was carried out on dry lyophilized AgNPs coated on a carbon film. High resolution transmission electron microscopy (HRTEM) imaging analysis was used for the size and shape confirmation of NPs using a TECNAI G2 (TEM) Electron Microscope (FEI company, Thermo Fisher Scientific, St. Bend, OR, USA) equipped with LaB6 filament at a point-resolution of 0.194 nm, lattice-resolution of 0.14 nm, tilt angle of ±60° and an accelerating voltage of 200 kV. The measurements were carried out by placing a single drop of nanoparticle solution on a carbon-coated Cu grid. The lattice spacing (d-spacing) was measured by calculating the distance between atomic planes of single NP in an enlarged HRTEM microscopic view using ImageJ software version 1.8.0_172 (NIH, Bethesda, MD, USA). The crystallinity of the sample was also evaluated by selected area electron diffraction (SAED) pattern generated by HRTEM.

### 2.3. Nematicidal Potential of GRAgNPs

#### 2.3.1. Culturing of Nematodes

A pure culture of an Indian isolate of *M. incognita* was obtained from Department of nematology, CCS HAU, Hisar, India. This culture was multiplied on the susceptible *Solanum melongena* Linn. (var. HLB-12) plants obtained from Department of vegetable science, CCS HAU, Hisar, India under green house conditions. After 35 days, the egg masses were collected manually from the root galls of infected plants. The freshly hatched juveniles (J2s) were extracted from these egg masses using the modified Baermann funnel technique [20].

#### 2.3.2. Hatching Inhibition

The egg masses were inoculated with different concentrations (0.5, 1, 2, 4, 6, 8, 10, 25, 50 and 75 ppm) of GRAgNPs along with standard checks [CAgNPs, VP, RE and double distilled water (DDW)] in six-well tissue culture plates (Tarsons products limited, Kolkata, India). In each well, two egg masses along with 1500 μL solution of each concentration (six replicates each) were inoculated and the plates were kept at room temperature. Observations on larvae hatching were recorded after three days of inoculation.

#### 2.3.3. Mortality Assay

Mortality assay using different treatments (0.5, 1, 2, 4, 6, 8, 10, 25, 50 and 75 ppm) of GRAgNPs along with standard checks (CAgNPs, VP, RE, and DDW), were conducted on freshly hatched J2s. The J2s were collected in 1.5 mL micro-centrifuge tubes and washed 3–4 times with DDW at 6000 rpm for 3 min. Aqueous suspension was standardized to 100 ± 3 nematodes per 10 μL. In a six-well tissue culture plate, 100 nematodes along with 1500 μL solution of each concentration were inoculated in each well and incubated at 27 °C (six replicates for each concentration).

The live larvae remained curled while the straightened ones were dead. After 12 and 24 h of treatment, observations were calculated using the following equation:

Mortality = (N_t_/N°) × 100 (1)

where, N_t_—Number of nematodes that died after each interval of time, and N°—The total number of nematodes inoculated.

The median lethal dose (LC50) for various concentrations of GRAgNPs, CAgNPs, VP, and RE were calculated and subjected to analysis of variance (ANOVA) using GraphPad Prism version 9 [21]. The obtained lowest LC50 was used for the selection of required concentration of GRAgNPs for the feeding experiment as well as for gene expression analysis.

#### 2.3.4. Uptake of GRAgNPs by J2s

The uptake of GRAgNPs was monitored by labeling them with fluorescein isothiocyanate (FITC). A 5 μL solution of FITC (5 mg/mL in DMSO) was mixed with 1 mL of GRAgNPs (1 ppm) and vortexed. To these FITC bound GRAgNPs, neurotransmitter octopamine solution (50 mM) was added. The mixture was fed to freshly hatched J2s in 1.5 mL micro-centrifuge tubes and kept on a Rotaspin (Tarsons, India) for 4 h at 12 rpm [22]. In the same manner, FITC bound CAgNPs, VP, and RE were fed to the J2s. J2s kept in DDW were fed with FITC alone. The J2s were visualized using fluorescent microscope (Zeiss Axioimager M2) at 200x magnification with an exposure period of 2.05 s at λ_excitation_ = 480 ± 10 nm and λ_emission_ = 520 ± 10 nm.

### 2.4. Effect of GRAgNPs on Differential Expression of Selected Candidate Genes

#### 2.4.1. Designing of Primers

Ten genes involved in DNA damage repair (*xpa-1*, *nth-1*) [23], detoxification and antioxidant pathway (*gst-n*, *ugt*, *skn-1*, *cpr-1*, *sod-3*, *mev-1*, *cyp-450*, and *dhs-23*) [24,25,26] were selected on the basis of available literature. Protein sequences of all ten identified genes were retrieved from *Caenorhabditis elegans* database and aligned with the genome resource of *M. incognita* [27] for the discerning of respective homologous protein-coding contigs. The protein- coding domains for the target genes were identified by using the NCBI domain finder software [28]. Primers were designed for the respective genes using IDT software [29] and synthesized commercially (Sigma, USA) (Table S1 in the Supplementary Materials).

#### 2.4.2. Total RNA Isolation and cDNA Synthesis

Freshly hatched J2s were collected in 1.5 mL micro-centrifuge tubes and washed 3–4 times with sterile DDW by centrifugation at 6000 rpm for 3 min. The total RNA was extracted from approximately 5000 nematodes using GeneJET RNA Purification Kit (Thermo Scientific, USA). The quality and quantity of total RNA were estimated by using NanoDrop 2000/2000c (Thermo Scientific, USA). cDNA synthesis from total RNA (300 ng) was carried out using the Verso cDNA synthesis kit (Thermo Scientific, USA).

#### 2.4.3. Expression Analysis of Target Genes

The expression of selected genes was investigated to explore the effect of GRAgNPs in comparison to CAgNPs and DDW. Freshly hatched J2s were fed with 1 ppm of both GRAgNPs and CAgNPs and kept at room temperature for 4 h at 12 rpm on a Rotaspin (Tarsons, India) along with DDW. Quantitative Real-time PCR (qRT-PCR) was performed using SYBER Green technology in BIO-RAD thermal cycler (BIO-RAD, Hercules, CA, USA). A master mix for each of the samples was prepared by adding iTaq^TM^ Universal SYBR Green Supermix (BIO-RAD, Hercules, CA, USA); 1.5 ng of cDNA and 750 nM each of the gene specific primers added to a final volume of 10 μL. The amplification reactions were carried out with a hot start of 95 °C for 4 min, followed by 40 cycles of 95 °C for 15 s and 62 °C for 1 min. Gene expression of non-treated nematodes was used as a base for comparison. *18s rRNA* was taken as an internal reference gene and also, two biological and three technical replicates were used for each of the samples. The mean CT values were taken for calculating the fold change in expression (2^−∆∆CT^) [30] and subjected to analysis of variance (ANOVA) using GraphPad Prism version 9.1.1 (225) (GraphPad Company, San Diego, CA, USA).

## 3. Results and Discussion

### 3.1. Synthesis and Characterization of GRAgNPs

**Figure 1:**
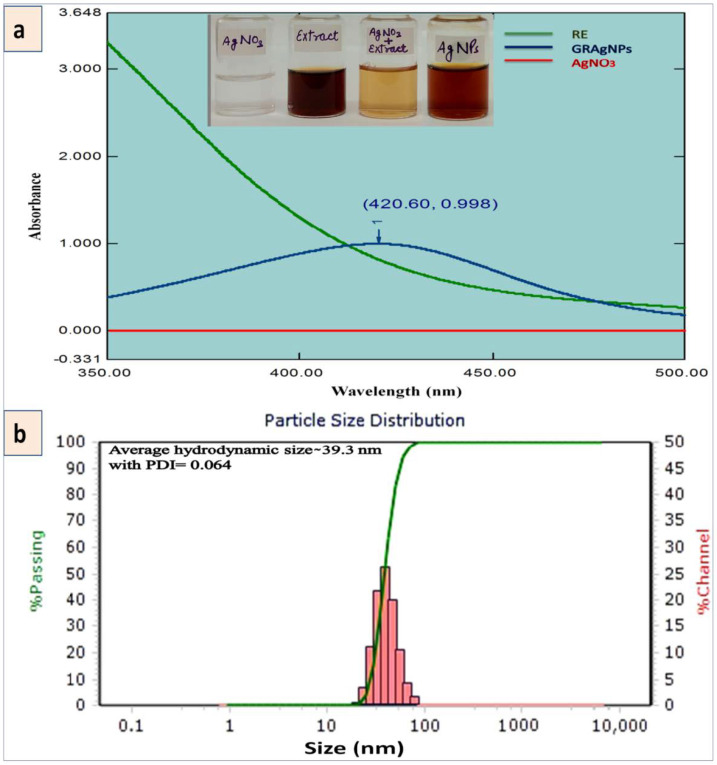
Synthesis of silver nanoparticles using aqueous root extract of *Glycyrrhiza glabra* (GRAgNPs). (**a**) UV-Vis absorption spectra of the aqueous root extract of *G. glabra* (RE), GRAgNPs and AgNO_3_ with inset showing visual color transition on mixing the RE with AgNO_3_ solution. (**b**) Particle size distribution of colloidal GRAgNPs.

The biosynthesis of GRAgNPs was ascertained by the visual color transition of the mixture of RE and AgNO_3_ from light yellow to colloidal brown. Figure 1a illustrates the color change due to biotransformation of Ag^+^ ions to Ag^0^ leading to the synthesis of GRAgNPs. GRAgNPs exhibited a strong surface plasmon resonance (SPR) peak at 420.60 nm with absorbance 0.998 while RE and AgNO_3_ did not exhibit any peaks. The size and spherical shape were confirmed by the SPR peak at a lower wavelength, which is in agreement with previous studies on the synthesis of GRAgNPs where spherical NPs of size 5–46 nm were found to be having SPR peaks from 404 to 445 nm [31,32,33,34]. High absorbance (~1) pointing towards the higher concentration of synthesized GRAgNPs in terms of their number [10].

The average hydrodynamic diameter was observed as 39.30 nm with a distribution range of 26.04–62.60 nm (Figure 1b). The PDI value was recorded as 0.0643 confirming the colloidal monodispersive nature of GRAgNPs and hence, the single intense peak was obtained. The stability of synthesized NPs was assessed by the zeta potential of −35.7 mV at 10 kV field-strength with conductivity of 106 uS/cm and mobility of 2.79 u/s/V/cm. The above 30 mV negative value of attained zeta potential affirmed the electrical stability of synthesized NPs due to repulsion among particles and no agglomeration as the phyto-constituents of RE (flavonoids, saponins, polyphenols, amines, proteins, sugars etc.) provided them stability by acting as natural reducing and capping agents. These findings were in consonance as reported by Kotakadi and co-workers [33], where the average hydrodynamic size of GRAgNPs of 41.8 nm with a distribution range of 33.4–74.3 nm and zeta potential of −34.1 mV were reported.

**Figure 2:**
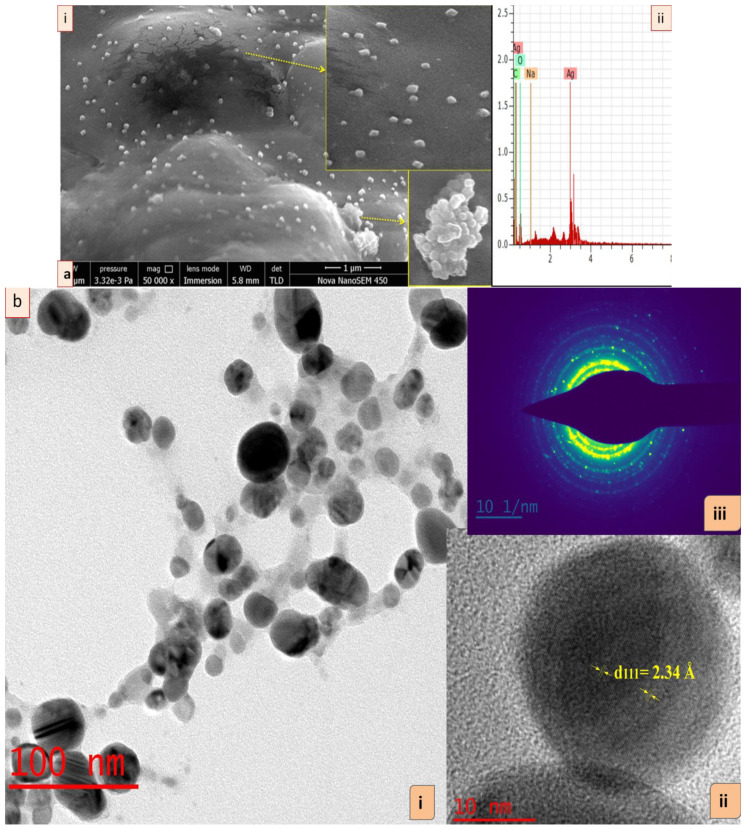
Characterization of silver nanoparticles synthesized using aqueous root extract of *G. glabra* (GRAgNPs). (**a**) (i) Field emission scanning electron micrograph of GRAgNPs at 50 kx magnification, (ii) Energy Dispersive X-ray spectrum of synthesized GRAgNPs, (**b**) HRTEM micrograph of GRAgNPs (i) spherical shape of NPs, (ii) single spherical GRAgNP and (iii) Ring SAED pattern of polycrystalline GRAgNPs. Insets showing enlarged portions.

The FE-SEM analysis exhibited the surface and shape of synthesized GRAgNPs. These were oval, cuboidal and spherical in shape and also coalesced to nanoclusters during scanning. SEM micrograph exhibited heterogeneity in shapes of well-distributed GRAgNPs as depicted in enlarged side panels (Figure 2a, panel (i)). Further confirmation of specific shape and size-distribution was undertaken by HRTEM analysis. For elemental analysis of GRAgNPs, Energy Dispersive X-ray (EDX) was used and the spectrum exhibited a strong characteristic signal peak at 3 keV confirming the presence of metallic AgNPs (Figure 2a, panel (ii)). The quantitative results of EDX illustrated a yield of 40.88 weight % of elemental silver in L-series of X-ray.

**Figure 3:**
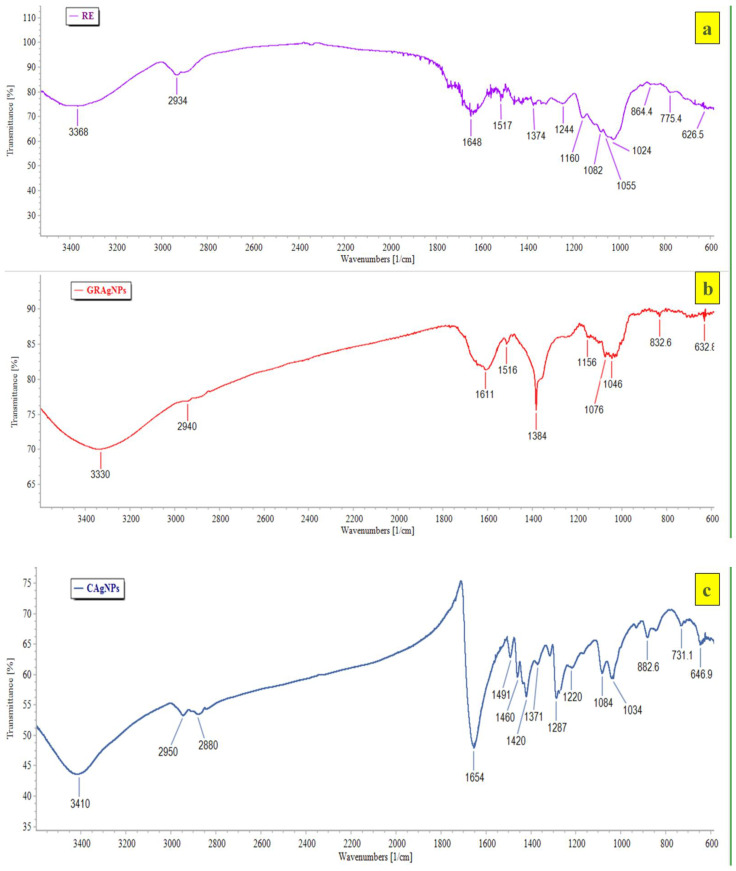
FTIR profile of (**a**) aqueous root extract of *G. glabra* (RE), (**b**) silver nanoparticles synthesized using aqueous root extract of *G. glabra* (GRAgNPs) and (**c**) commercial silver nanoparticles (CAgNPs) depicting spectral bands at various positions.

The HRTEM images of GRAgNPs showed that NPs are almost spherical in shape with uniform size distribution (Figure 2b, panel (i)). The clear and similar lattice spacing (d-spacing) between atomic planes was observed as 2.34 Å (Figure 2b, panel (ii)). The polycrystalline nature of spherical NPs was confirmed by the clear and uniform concentric rings as shown in Selected Area (Electron) Diffraction (SAED) pattern (Figure 2b, panel (iii)). The mean particle size obtained was 20.017 ± 6.79 with a range of 9.61–34.735 nm. HRTEM size was found to be in concurrence with prior reports on green synthesis of GRAgNPs exhibiting size distribution of 20–30 nm [31], 7–45 nm [32], 10–45 nm [33] and 46 nm [34]. The obtained lattice spacing distance is in agreement with the published value of miller indices (h k l) of (1 1 1) d-spacing of face-centered cubic (FCC) silver [35]. The FTIR spectral analysis of GRAgNPs depicted that the biomolecules present in RE lead to the shifting in various peaks due to the interaction of their functional groups within the synthesized NPs. The FTIR profile of RE showed various peaks at 3368, 2934, 1648, 1517, 1374, 1244, 1160, 1082, 1055, 1024, 864.4, 775.4, 626.5 cm^−1^, whereas the GRAgNPs showed spectral bands at following positions 3330, 2940, 1611, 1516, 1384, 1156, 1076, 1046, 832.6, 632.8 cm^−1^ (Figure 3a,b). In addition, the FTIR profile of CAgNPs showed various peaks at 3410, 2950, 2880, 1654, 1491, 1460, 1420, 1371, 1287, 1220, 1084, 1034, 882.6, 731.1, 646.9 cm^−1^, which were in concurrence with those exhibited by polyvinylpyrrolidone (PVP) coated CAgNPs dispersed in ethylene glycol [36,37] (Figure 3c).

FTIR spectral analysis of GRAgNPs revealed distinctive broad spectral bands at 3570–3200 cm^−1^ that are characteristic to the –OH stretching vibration of hydroxyl functional groups in poly-phenols and –NH stretching vibration in primary and secondary amines of amino acids, peptides and proteins present in RE. The narrow band observed at below 3000 cm^−1^ is characteristic of aliphatic compounds. The peak at the range of 1690–1575 cm^−1^ could be attributed to the –C=N– stretching of open chain imino compounds, and –N=N– stretching of open chain azo compounds. The peaks at the range of 1610–1550/1420–1300 cm^−1^, 1410–1310 cm^−1^, 1100–1000 cm^−1^, 890–820 cm^−1^, 705–570 cm^−1^ and 660–630 cm^−1^ may be credited to stretching of the RCOO^−^ group of carboxylic acid salts, –OH bending vibration of hydroxyl functional groups present in phenols or tertiary alcohols, phosphate ion, –C–O–O stretching of peroxides, –C–S stretching of disulphides, and thioethers, respectively. The FTIR spectrum revealed that the major bioactive compounds involved in biotransformation of Ag^+^ ions to Ag^0^, and capping and stabilization of GRAgNPs might be flavonoids (liquiritin, liquiritigenin, glabridin), saponins (glycyrrhizic acid, glycyrrhetinic acid), polyphenols (coumeric acid, apigenin), coumarins, amines, proteins and sugars. [38].

### 3.2. Bio-Efficacy of GRAgNPs

#### 3.2.1. Hatching Inhibition

**Figure 4:**
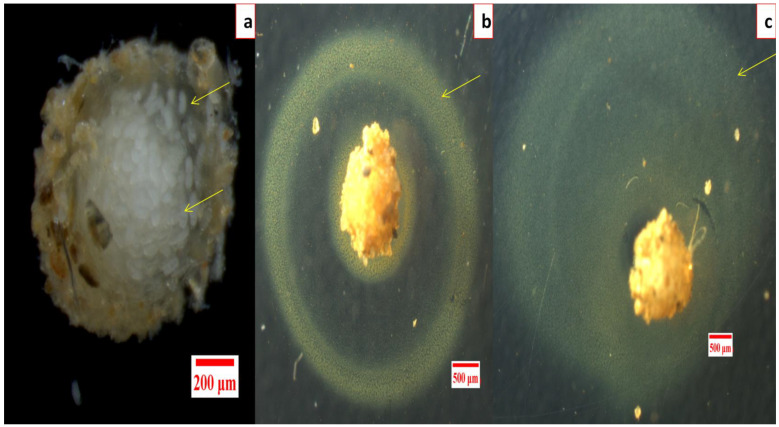
Effect of silver nanoparticles synthesized using aqueous root extract of *G. glabra* (GRAgNPs) on hatching inhibition in *M. incognita,* (**a**) healthy egg mass, (**b**) GRAgNPs treated egg mass after 24 h, (**c**) after 48 h of the treatment.

No hatching inhibition was observed in DDW treated egg masses as 56.5 ± 6.09 live J2s after 24 h and 145.33 ± 4.63 live J2s after 48 h were observed in each well (For reference, a healthy egg mass can be seen in Figure 4a).

Similarly, in RE treated egg masses, no hatching inhibition was seen even at 75.0 ppm as 51.17 ± 6.62 live J2s after 24 h and 146.67 ± 6.12 J2s after 48 h were observed. Complete hatching inhibition in egg masses was observed after 48 h of incubation with the lowest dose of 10.0 ppm of GRAgNPs, 50.0 ppm of CAgNPs, and 25.0 ppm of VP, respectively (Table S2 in the Supplementary Materials). However, a peculiar yellow ring around the gelatinous matrix of entire egg mass was observed only in the case of GRAgNPs at 75.0 ppm after 24 h of the treatment (Figure 4b,c).

#### 3.2.2. Mortality Assay

**Figure 5:**
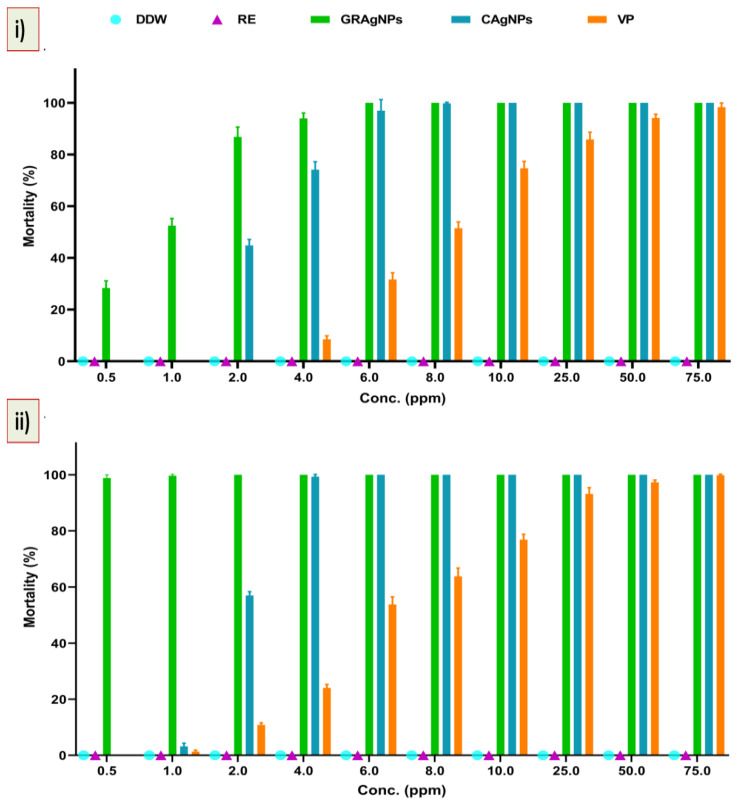

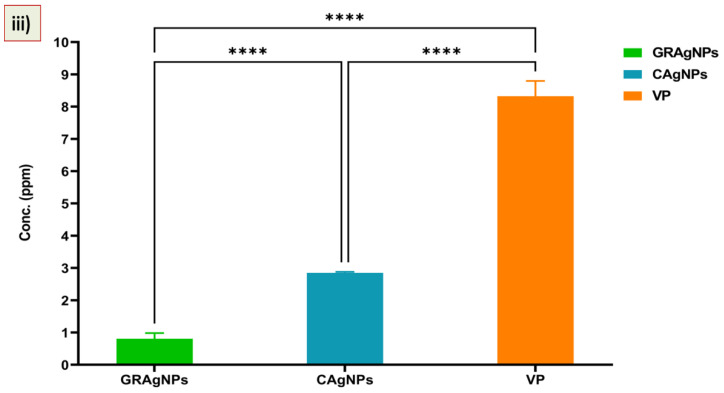
Mortality (%) and response of second stage juveniles (J2s) to GRAgNPs treatment. (i) % mortality after 12 h, (ii) % mortality after 24 h of treatment, (iii) Graph showing median lethal dose (LC-50) of GRAgNPs, CAgNPs, and VP calculated using one-way ANOVA with Turkey’s multiple comparisons test at 95% CI and α=0.05 (where **** = *p* < 0.0001). Columns are mean of 6 replicates; error bars represent the standard error. [Where, DDW = double distilled water, RE = aqueous root extract of *G. glabra*, GRAgNPs = silver nanoparticles synthesized using aqueous root extract of *G. glabra*, CAgNPs = commercial silver nanoparticles, and VP = commercial nematicide, Velum prime].

It was evident from the data that mortality rate was dependent upon the type of treatment, concentration, and exposure time. No nematicidal activity of RE was observed, whereas 100 % mortality was recorded at the lowest dose of 6.0 ppm, and 10.0 ppm in case of GRAgNPs, and CAgNPs, respectively, after 12 h of treatment. The commercial nematicide (VP) caused 98.0 % mortality at a relatively very high concentration of 75.0 ppm (Figure 5, panel (i)). It was observed that on increasing exposure time of treatment from 12 h to 24 h, 100% mortality was achieved at relatively lower concentrations. After 24 h, GRAgNPs exhibited a relatively very high mortality of 98.83%, 99.67% and 100% at 0.5 ppm, 1.0 ppm, and 2.0 ppm, respectively, whereas VP and CAgNPs did not exhibit any mortality at 0.5 ppm (Figure 5, panel (ii)). In contrast to GRAgNPs, VP and CAgNPs showed very little mortality, 1.33%, and 3.17% at 1.0 ppm, and 10.83%, and 57.0% at 2.0 ppm, respectively (Table S3 in the Supplementary Materials). After 12 h doses from 0.5 to 6.0 ppm were used to calculate LC-50 for GRAgNPs, CAgNPs and VP. LC-50 for GRAgNPs, CAgNPs and VP was found to be 0.805 ± 0.177, 2.849 ± 0.032, 8.325 ± 0.473 ppm, respectively (Figure 5, panel (iii)). GRAgNPs were observed to be the most efficient at 0.805 ppm to kill 50.0% of J2s, which is approximately 1/3rd of LC-50 of CAgNPs, and 1/10th of VP. The ordinary one-way ANOVA with Turkey’s multiple comparisons test showed statistically significant difference in median LC-50 doses in accordance with the treatment type, where F (2, 15) = 1061, *p* < 0.0001, R^2^ = 0.9930 at 95% confidence interval and α = 0.05 were observed (Figure 5, panel (iii)).

The effective dose required to kill the J2s was found to be very low, whereas the use of higher concentration of AgNPs at 30 to 150 ppm [39], 20–40 ppm [10], and 25–90% [40], and 125–1000 mgL^−1^ [15] has been reported. Our results are in agreement with reports by Baronia and co-workers, where 100% mortality was achieved at 2 ppm after 24 h of AgNPs treatment [41].

#### 3.2.3. Uptake of GRAgNPs by J2s

**Figure 6:**
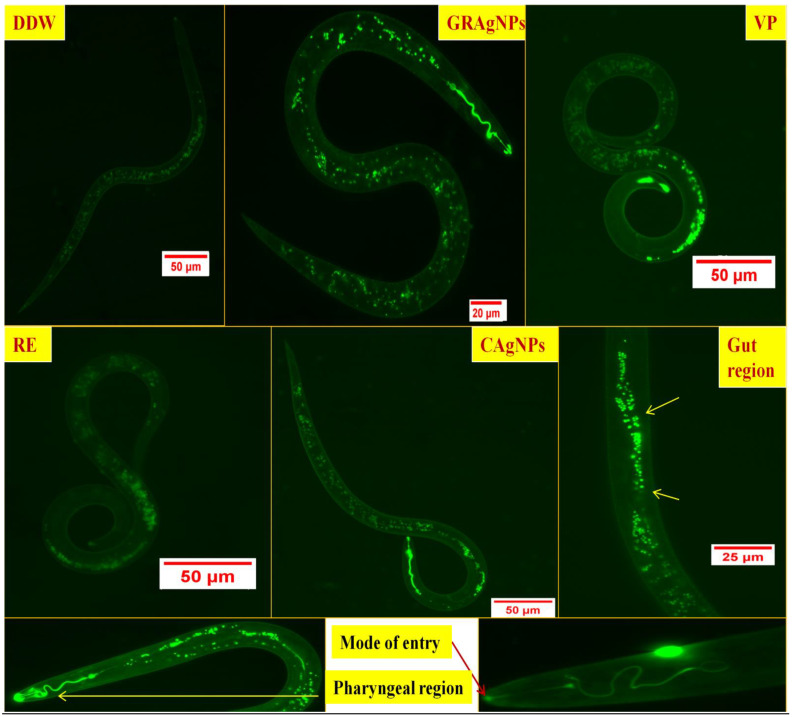
Fluorescent micrograph of uptake of GRAgNPs, CAgNPs, VP, and RE in *M. incognita* J2s. [Where, DDW = double distilled water, GRAgNPs = silver nanoparticles synthesized using aqueous root extract of *G. glabra*, VP = commercial nematicide, Velum prime, RE = aqueous root extract of *G. glabra*, and CAgNPs = commercial silver nanoparticles].

Pharyngeal pumping in nematodes play a crucial role in intake of particles from surrounding and their transport inside the intestine, whereas intestinal epithelium helps in their translocation to other locations via pseudocoelomic fluid [42]. The fluorescent micrograph showed that FITC labeled GRAgNPs fluoresced with high intensity primarily in the intestinal region, whereas low intensity auto-fluorescence was observed in untreated control J2s. The highest fluorescent intensity was observed in GRAgNPs as compared to CAgNPs and VP, whereas low-intensity auto-fluorescence was seen in DDW and RE (Figure 6).

### 3.3. Effect of GRAgNPs on Differential Expression of Selected Genes

**Figure 7:**
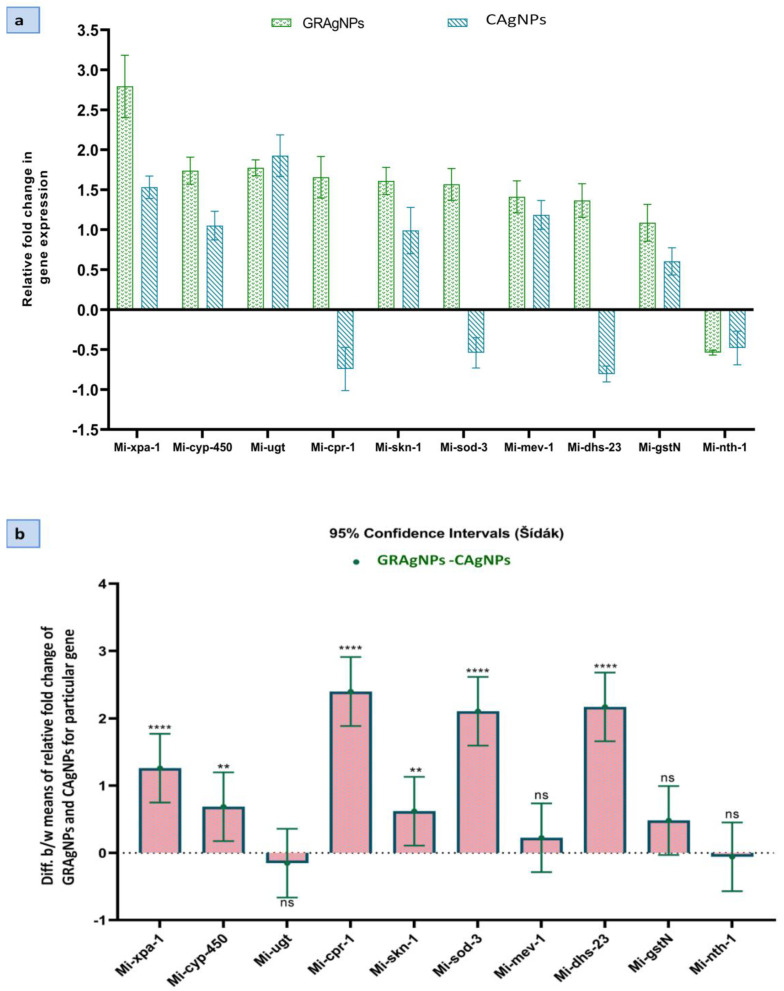
Comparative expression analysis of target genes in *M. incognita* exposed to silver nanoparticles synthesized using aqueous root extract of *G. glabra*, GRAgNPs (1 ppm) and, commercial silver nanoparticles, CAgNPs (1 ppm) for 4 hrs. (**a**) Relative fold change in expression. (**b**) 95% CI of difference between means of relative fold change of GRAgNPs and CAgNPs for a particular gene calculated using 2-way ANOVA with Šídák’s multiple comparisons test at α = 0.05 (where **** = *p* < 0.0001, ** = *p* < 0.01 and ns = non-significant).

J2s larvae treated with GRAgNPs showed up-regulation of nine genes, including *skn-1*, *mev-1*, *sod-3*, *dhs-23*, *cyp-450*, *xpa-1*, *cpr-1*, *gst-n*, and *ugt* (1.09–2.79 folds), and down-regulation of only one gene, *nth-1* (0.54 folds). CAgNPs caused up-regulation (0.60–1.93 folds) of six genes (*skn-1*, *mev-1*, *cyp-450*, *xpa*, *gst-n*, and *ugt*), and down-regulation (0.48–0.80 folds) of four genes (*sod-3*, *dhs-23*, *cpr-1* and *nth-1*) (Figure 7a).

Gene expression can be modulated under different conditions such as survival, growth, reproduction, stress, toxicity, etc. To overcome the oxidative stress, expression of detoxifying and antioxidant genes is enhanced in nematodes [17,23]. The up-regulation of oxidative stress response genes such as *gst-n*, *ugt* and *cpr-1* exhibited that the reactive oxygen species (ROS) were scavenged by glutathione transferase (GST), glucuronosyl transferase, and peptidase activities, respectively in response to GRAgNPs created oxidative stress. The elevated expression (2.4 times) of *cpr-1* in GRAgNPs treated J2s larvae revealed the higher oxidative stress caused by GRAgNPs in comparison to CAgNPs, which was highly significant at *p* < 0.0001 and 95% CI.

Genes such as *mev-1*, *sod-3*, *dhs-23*, and *cyp-450,* showed enhanced expression during the oxidative stress. The approximately two-fold increase in expression of *sod-3* and 2.17-fold increase in the activity of *dhs-23* in the case of GRAgNPs treated J2s larvae as compared to CAgNPs, suggested the intensified removal of superoxide radicals and enhanced reductase and dehydrogenase activities. The enhanced *cyp-450* expression pointed towards strong response to xenobiotic stimulus in the form of exposure to AgNPs. Product of *xpa*-1 interacts directly with DNA substrates as well as proteins involved in damage recognition to gap-filing synthesis in the nuclear excision pathway [43]. Enhanced expression of *xpa*-1 (2.8-fold) was observed in GRAgNPs treated J2s as compared to CAgNPs (1.5-fold). This showed the higher nanotoxic impact of GRAgNPs towards the nematode in contrast to CAgNPs. All these findings were statistically relevant at 95% CI, *p* < 0.001 and *p* < 0.01. In our study, the downregulation of *nth-1* activity in both GRAgNPs and CAgNPs was observed.

The ordinary two-way ANOVA showed that the Dosage x Relative fold change in gene expression interaction accounted for 19.55% of total variance, whereas dosage for 22.68% and relative fold change in gene expression for 54.92% at *p* < 0.0001 and α = 0.05 and the effects were considered extremely significant. The Šídák’s multiple comparisons post hoc test compared the means of both GRAgNPs and CAgNPs on the expression of a particular gene at 95% CI and α = 0.05 (Figure 7b). Moreover, 95% CI of difference was found to be extremely significant for *xpa-1*, *cpr-1*, *sod-3*, and *dhs-23,* highly significant for *skn-1* and *cyp-450,* and non-significant for *mev-1*, *gst-n*, *ugt* and *nth-1*. All these differences of treatment means are large enough to be scientifically relevant.

Effect of GRAgNPs on differential expression of selected candidate genes showed that the upregulation of these stress responsive genes could be due to the nanotoxicity towards the nematode by causing oxidative stress. The mechanism of action of green AgNPs against the nematode needs further investigation. Most probably, this nanotoxicity could be due to the disruption of the cellular mechanisms including response to oxidative stress, DNA damage repair, detoxification, ATP synthesis and membrane permeability [44] etc.

In contrast to AgNPs, chemical nematicides such as VP inhibit ATP synthesis by blocking the succinate dehydronase (SDH) enzyme targeting the mitochondrial respiratory chain. Thus, affecting cellular respiration and leading to the immobilization of nematode and then its death shortly after. This is a fluopyram-based chemical formulation having hazardous impact on human health as well as on the environment and must be used with all safety precautions at various levels, including aquatic and terrestrial ecosystems, operator, storage, and disposal [45]. For the development of target specific nano-nematicidal formulation(s) with their controlled release in the environment, a reliable mechanistic approach is needed covering all the domains.

## 4. Conclusions

In the present study, stable GRAgNPs were synthesized using aqueous root extract of *G. glabra* (var. HM-1), characterized and their efficacy was checked against root-knot nematode, *M. incognita*. The active uptake of FITC labeled GRAgNPs and their effect on expression of selected genes involved in DNA damage repair, detoxification, and antioxidant pathway were also studied. The findings revealed that RE did not inhibit the egg-hatching while no hatching was observed after 48 h of treatment with as low as 10.0 ppm for GRAgNPs, 25.0 ppm for VP, and 50.0 ppm for CAgNPs. Similarly, no mortality was caused by RE, whereas 100 per cent mortality was observed after 12 h of treatment with as low as 6.0 ppm of GRAgNPs, and 10.0 ppm of CAgNPs. The commercial nematicide (VP) caused 98 per cent mortality at a relatively very high concentration (75.0 ppm). The LC-50 for GRAgNPs, CAgNPs and VP was found to be 0.805 ± 0.177, 2.849 ± 0.032, 8.325 ± 0.473 ppm, respectively at *p* < 0.0001, R^2^ = 0.9930, 95% CI and α = 0.05. The LC-50 for CAgNPs and VP were approx. 3.5 and 10 times higher than that of GRAgNPs, respectively. The FITC-labeled GRAgNPs fluoresced with high intensity primarily in the gut region of the nematode. In addition, nine genes, except *nth-1*, were upregulated on exposure (4 h) to 1.0 ppm of GRAgNPs while six genes (*skn-1, mev-1, cyp-450, xpa, gst-n,* and *ugt*) were upregulated in case of CAgNPs treatment. The upregulation of these genes could be due to the nanotoxicity caused by the GRAgNPs towards the nematode by causing oxidative stress. On the basis of above results, we can conclude that the green synthesized GRAgNPs can be utilized as an efficacious and promising alternative to chemical nematicides and chemically synthesized CAgNPs as they exhibited lower LC-50, higher mortality and nanotoxic effect against *M. incognita*. GRAgNPs could be used in the development of target specific nano-nematicidal formulation(s) with their controlled release in the environment. Moreover, the green route of synthesis requires less overall chemical usage and produces no toxic chemical residues, which proves to be an efficacious tool in providing a green and sustainable alternative to the highly toxic chemical nematicides. Thus, GRAgNPs could be used for controlling nematode infestations which cause severe crop yield losses globally. To the best of our knowledge, it is the first report on this plant in the context of nematicidal potential of GRAgNPs against *M. incognita* and their impact on the expression of selected genes involved in oxidative stress and DNA damage repair.

## 5. Future Outlook

Chemical nematicides cause residual toxicity and environmental pollution. Moreover, their extensive use results in resistance in nematodes against them. Therefore, an alternative green economical approach is needed for their management. Green AgNPs possess great antimicrobial potential and can be used for this purpose. A holistic approach is needed to comprehend the behavioral and functional aspects of green AgNPs both at in-vitro and in-vivo levels. Further research is required to elucidate their impact towards other living systems and crops.

## Data Availability

Data are available on request.

## Supplementary Materials

The following supporting information can be downloaded at: https://www.mdpi.com/2079-4991/12/17/2966/s1, Table S1: List of selected genes and gene specific primers. Table S2: Effect of RE, GRAgNPs, CAgNPs, and VP on hatching-inhibition after 24 h and 48 h of treatment. Table S3: Effect of RE, GRAgNPs, CAgNPs, and VP on mortality (%) in J2s after 12 h and 24 h of treatment

## References

[1] Han H.J., Nwagwu C., Anyim O., Ekweremadu C., Kim S. (2021). COVID-19 and cancer: From basic mechanisms to vaccine development using nanotechnology. Int. Immunopharmacol..

[2] Mohammad Z.H., Ahmad F., Ibrahim S.A., Zaidi S. (2022). Application of nanotechnology in different aspects of the food industry. Discov. Food.

[3] Maghsoudi A.S., Hassani S., Mirnia K., Abdollahi M. (2021). Recent Advances in Nanotechnology-Based Biosensors Development for Detection of Arsenic, Lead, Mercury, and Cadmium. Int. J. Nanomed..

[4] Jaskulski D., Jaskulska I., Majewska J., Radziemska M., Bilgin A., Brtnicky M. (2022). Silver Nanoparticles (AgNPs) in Urea Solution in Laboratory Tests and Field Experiments with Crops and Vegetables. Materials.

[5] Nazari N., Kashi F.J. (2020). A novel microbial synthesis of silver nanoparticles: Its bioactivity, Ag/Ca-Alg beads as an effective catalyst for decolorization Disperse Blue 183 from textile industry effluent. Sep. Purif. Technol..

[6] Mustapha T., Misni N., Ithnin N.R., Daskum A.M., Unyah N.Z. (2022). A Review on Plants and Microorganisms Mediated Synthesis of Silver Nanoparticles, Role of Plants Metabolites and Applications. Int. J. Environ. Res. Public Health.

[7] Khan M., Khan A.U., Bogdanchikova N., Garibo D. (2021). Antibacterial and antifungal studies of biosynthesized silver nanoparticles against plant parasitic nematode *Meloidogyne incognita*, plant pathogens *Ralstonia solanacearum* and *Fusarium oxysporum*. Molecules.

[8] Ahmed S., Ahmad M., Swami B.L., Ikram S. (2015). A review on plants extract mediated synthesis of silver nanoparticles for antimicrobial applications: A green expertise. J. Adv. Res..

[9] Kaabipour S., Hemmati S. (2021). A review on the green and sustainable synthesis of silver nanoparticles and one-dimensional silver nanostructures. Beilstein J. Nanotechnol..

[10] Fouda M.M., Abdelsalam N.R., Gohar I.M.A., Hanfy A.E., Othman S.I., Zaitoun A.F., Allam A.A., Morsy O.M., El-Naggar M. (2020). Utilization of High throughput microcrystalline cellulose decorated silver nanoparticles as an eco-nematicide on root-knot nematodes. Colloids Surf. B Biointerfaces.

[11] Milligan S.B., Bodeau J., Yaghoobi J., Kaloshian I., Zabel P., Williamson V.M. (1998). The root knot nematode resistance gene Mi from tomato is a member of the leucine zipper, nucleotide binding, leucine-rich repeat family of plant genes. Plant Cell.

[12] Ghareeb R.Y., El-Din N.G.E.-D.S., El Maghraby D.M., Ibrahim D.S., Abdel-Megeed A., Abdelsalam N.R. (2022). Nematicidal activity of seaweed-synthesized silver nanoparticles and extracts against *Meloidogyne incognita* on tomato plants. Sci. Rep..

[13] Kalaiselvi D., Mohankumar A., Shanmugam G., Nivitha S., Sundararaj P. (2018). Green synthesis of silver nanoparticles using latex extract of Euphorbia tirucalli: A novel approach for the management of root knot nematode, Meloidogyne incognita. Crop Prot..

[14] Rani K., Devi N., Saharan V., Kharb P. (2021). *Glycyrrhiza glabra*: An insight to nanomedicine. J. Nanosci. Nanotechnol..

[15] Nassar A.M. (2016). Research Article Effectiveness of Silver Nano-particles of Extracts of *Urtica urens* (Urticaceae) Against Root-knot Nematode Meloidogyne incognita. Asian J. Nematol..

[16] Abbassy M.A., Abdel-Rasoul M.A., Nassar A.M., Soliman B.S. (2017). Nematicidal activity of silver nanoparticles of botanical products against root-knot nematode, Meloidogyne incognita. Arch. Phytopathol. Plant Prot..

[17] Lim D., Roh J.Y., Eom H.J., Choi J.Y., Hyun J., Choi J. (2012). Oxidative stress-related PMK-1 P38 MAPK activation as a mechanism for toxicity of silver nanoparticles to reproduction in the nematode *Caenorhabditis elegans*. Environ. Toxicol. Chem..

[18] Sharma V., Katiyar A., Agrawal R.C., Mérillon J.M., Ramawat K. (2018). Glycyrrhiza glabra: Chemistry and pharmacological activity. Sweeteners. Reference Series in Phytochemistry.

[19] El-Saber Batiha G., Beshbishy A.M., El-Mleeh A., Abdel-Daim M., Devkota H.P. (2020). Traditional uses, bioactive chemical constituents, and pharmacological and toxicological activities of *Glycyrrhiza glabra* L. (Fabaceae). Biomolecules.

[20] Staniland L.N. (1954). A modification of the Baermann funnel technique for the collection of nematodes from plant material. J. Helminthol..

[21] One-Way ANOVA Followed by Dunnett’s Multiple Comparisons Test Was Performed Using GraphPad Prism Version 9.1.1(225) for Windows, GraphPad Software, San Diego, CA, USA. www.graphpad.com.

[22] Bakhetia M., Charlton W., Atkinson H.J., McPherson M.J. (2005). RNA interference of dual oxidase in the plant nematode Meloidogyne incognita. Mol. Plant-Microbe Interact..

[23] Meyer J.N., Lord C.A., Yang X.Y., Turner E.A., Badireddy A.R., Marinakos S.M., Chilkoti A., Wiesner M.R., Auffan M. (2010). Intracellular uptake and associated toxicity of silver nanoparticles in *Caenorhabditis elegans*. Aquat. Toxicol..

[24] Wu C.W., Deonarine A., Przybysz A., Strange K., Choe K.P. (2016). The Skp1 homologs SKR-1/2 are required for the *Caenorhabditis elegans* SKN-1 antioxidant/detoxification response independently of p38 MAPK. PLoS Genet..

[25] Di R., Zhang H., Lawton M.A. (2018). Transcriptome analysis of *C. elegans* reveals novel targets for DON cytotoxicity. Toxins.

[26] Fontaine P., Choe K. (2018). The transcription factor SKN-1 and detoxification gene ugt-22 alter albendazole efficacy in *Caenorhabditis elegans*. Int. J. Parasitol. Drugs Drug Resist..

[27] Resources for Meloidogyne Incognita Genome. https://www6.inrae.fr/meloidogyne_incognita.

[28] NCBI Conserved Domain Search. https://www.ncbi.nlm.nih.gov/Structure/cdd/wrpsb.cgi.

[29] Primer Quest Desigen qPCR Assays: Type Equation Here. https://www.idtdna.com/pages/tools/primerquest.

[30] Livak K.J., Schmittgen T.D. (2001). Analysis of relative gene expression data using real-time quantitative PCR and the 2^−ΔΔCT^ method. Methods.

[31] Dinesh S., Karthikeyan S., Arumugam P. (2012). Biosynthesis of silver nanoparticles from *Glycyrrhiza glabra* root extract. Arch. Appl. Sci. Res..

[32] Sreelakshmy V., Deepa M.K., Mridula P. (2016). Green synthesis of silver nanoparticles from *Glycyrrhiza glabra* root extract for the treatment of gastric ulcer. J. Dev. Drugs.

[33] Kotakadi V.S., Gaddam S.A., Venkata S.K., Sarma P.V.G.K., Gopal D.V.R.S. (2016). Biofabrication and spectral characterization of silver nanoparticles and their cytotoxic studies on human CD34+ ve stem cells. 3 Biotech.

[34] Pandian N., Chidambaram S. (2017). Antimicrobial, cytotoxicty and anti cancer activity of silver nanoparticles from *Glycyrrhiza glabra*. Int. J. Pharm. Sci. Res..

[35] Wan H., Li C., Mahmud S., Liu H. (2021). Kappa carrageenan reduced-stabilized colloidal silver nanoparticles for the degradation of toxic azo compounds. Colloids Surfaces A Physicochem. Eng. Asp..

[36] Zhao T., Sun R., Yu S., Zhang Z., Zhou L., Huang H., Du R. (2010). Size-controlled preparation of silver nanoparticles by a modified polyol method. Colloids Surfaces A Physicochem. Eng. Asp..

[37] Leng Z., Wu D., Yang Q., Zeng S., Xia W. (2018). Facile and one-step liquid phase synthesis of uniform silver nanoparticles reduction by ethylene glycol. Optik.

[38] Nandiyanto A.B.D., Oktiani R., Ragadhita R. (2019). How to read and interpret FTIR spectroscope of organic material. Indones. J. Sci. Technol..

[39] Cromwell W.A., Yang J., Starr J.L., Jo Y.K. (2014). Nematicidal effects of silver nanoparticles on root-knot nematode in bermudagrass. J. Nematol..

[40] Hassan M.E., Zawam H.S., Nahas S.E.E., Desoukey A.F. (2016). Comparison study between silver nanoparticles and two nematicides against Meloidogyne incognita on tomato seedlings. Plant Pathol. J..

[41] Baronia R., Kumar P., Singh S.P., Walia R.K. (2020). Silver nanoparticles as a potential nematicide against. J. Nematol..

[42] Gonzalez-Moragas L., Roig A., Laromaine A. (2015). *C. elegans* as a tool for in vivo nanoparticle assessment. Adv. Colloid Interface Sci..

[43] Elsakrmy N., Zhang-Akiyama Q.-M., Ramotar D. (2020). The base excision repair pathway in the nematode *Caenorhabditis elegans*. Front. Cell Dev. Biol..

[44] Basso M.F., Lourenço-Tessutti I.T., Mendes R.A.G., Pinto C.E.M., Bournaud C., Gillet F.-X., Togawa R.C., de Macedo L.L.P., de Almeida Engler J., Grossi-de-Sa M.F. (2020). MiDaf16-like and MiSkn1-like gene families are reliable targets to develop biotechnological tools for the control and management of *Meloidogyne incognita*. Sci. Rep..

[45] Safety Data Sheet of Velum Prime. www.cropscience.bayer.co.uk/velumprimesds.

