# Correction: Rani et al. Nematicidal Potential of Green Silver Nanoparticles Synthesized Using Aqueous Root Extract of *Glycyrrhiza glabra*. *Nanomaterials* 2022, *12*, 2966

**DOI:** 10.3390/nano14070585

**Published:** 2024-03-27

**Authors:** Kanika Rani, Nisha Devi, Prakash Banakar, Pushpa Kharb, Prashant Kaushik

**Affiliations:** 1 Department of Molecular Biology, Biotechnology and Bioinformatics, Chaudhary Charan Singh Haryana Agricultural University, Hisar 125004, India; 2 Department of Nematology, Chaudhary Charan Singh Haryana Agricultural University, Hisar 125004, India; 3 Kikugawa Research Station, Yokohama Ueki, Kikugawa 439-0031, Japan

## Affiliation Correction

In the publication [1], there was an error regarding the affiliation of Prashant Kaushik. The affiliation “Instituto de Conservación y Mejora de la Agrodiversidad Valenciana, Universitat Politècnica de València, 46022 Valencia, Spain” has been removed per the institution’s request. The authors state that the scientific conclusions are unaffected. This correction was approved by the Academic Editor. The original publication has also been updated.
