# Supplementary material for: Nematicidal Potential of Green Silver Nanoparticles Synthesized Using Aqueous Root Extract of Glycyrrhiza glabra
Source: Nanomaterials (Basel). 2022 Aug 27;12(17):2966. doi: 10.3390/nano12172966 (PMC9458125; doi:10.3390/nano12172966)
Supplement: Supplementary file 1 [file nanomaterials-12-02966-s001.zip › nanomaterials-1817470-supplementary.pdf]

Table S1. List of selected genes and gene specific primers.

Sr. No	Gene (Accession No.)	Function of Gene	Primer Sequence (5'→3')	Tm (°C)	Amplicon Size (bp)
1	*Mi-18S rRNA* (HE667742)	Adenine dimethyltransferase activity and RNA binding activity	F:TCAACGTGCTTGTCCTACCCTGAA<br>R:TGTGTACAAAGGGCAGGGACGTAA	60	155
2	*Mi-cpr-1* (Minc3s02882g31948)	Cysteine-type endopeptidase activity	F:CTGCACAGGATCTGGTTATT<br>R:GTAGACTCACTGGTTCTTGTC	60	173
3	*Mi-cyp-450* (Minc3s00842g17994)	Oxidative stress defense	F:GAGCAATTAGTAGCCGTTGT<br>R:GGCAATGATGGCCTATCTG	60	188
4	*Mi-dhs-23* (Minc3s08123g41921)	Oxidoreductase activity	F:TCAATTAGCCGTGCCTTATC<br>R:CTGGGTTCACAGCATTAACA	60	188
5	*Mi-gst-N* (Minc3s00350g10772)	Glutathione Dehydrogenase (Ascorbate) activity	F:GATTGCTGTATTCCTCCACAAG<br>R:TCTTCTTCTGGCGTATCTGG	60	155
6	*Mi-mev-1* (Minc3s00018g01157)	Oxidative stress defense	F:GGTGGTCATCGAATTAGTGG<br>R:CGAATCCCATTGAGTGAGTG	60	194
7	*Mi-nth-1* (Minc3s00396g11575)	DNA repair mechanism	F:GCTGGCTGTTCAATTTCTTC<br>R:CAACACCTGGAAGTTCACAT	60	184
8	*Mi-skn-1* (Minc3s02028g27862)	Oxidative stress defense	F:CCTTAATGCAGGATGTTCGT<br>R:CCTCATCCCAATCTTCTTCATC	60	183
9	*Mi-sod-3* (Minc3s03341g33565)	Oxidative stress defense	F:GACTTTGGTTCTCTCCAAGG<br>R:CCCAAACGTCAATTCCAAAG	60	187
10	*Mi-ugt* (Minc3s02431g29998)	Detoxification	F:CACAGTCTTGAAGGAATTGGTG<br>R:TCTGAGACTTCTGGTTGAGA	60	182
11	*mi-xpa-1 (Minc3s00281g09312)*	DNA repair mechanism	F:CGATGCCTATCTGTGGAAAT<br>R:CTCCATAGCGTGGATTATGC	60	188

Table S2. Effect of RE, GRAgNPs, CAgNPs, and VP on hatching-inhibition after 24 h and 48 h of treatment.

Control/Treatment	RE		GRAgNPs		CAgNPs		VP	
**Conc. (ppm)**	**24 h**	**48 h**	**24 h**	**48 h**	**24 h**	**48 h**	**24 h**	**48 h**
DDW	56.50 ± 6.09	145.33 ± 4.63	56.50 ± 6.09	145.33 ± 4.63	56.50 ± 6.09	145.33 ± 4.63	56.50 ± 6.09	145.33 ± 4.63
0.5	54.50 ± 5.47	144.33 ± 3.56	9.17 ± 1.72	83.50 ± 6.75	7.33 ± 1.37	71.67 ± 5.05	52.17 ± 5.91	146.17 ± 5.56
1.0	49.33 ± 8.59	143.50 ± 6.47	6.33 ± 1.03	73.17 ± 4.12	6.67 ± 1.21	62.17 ± 5.56	47.50 ± 6.47	139.83 ± 6.11
2.0	56.50 ± 8.26	143.67 ± 2.80	1.00 ± 0.85	65.00 ± 4.74	5.33 ± 0.82	62.67 ± 1.86	48.50 ± 5.01	135.83 ± 3.76
4.0	54.50 ± 9.65	141.67 ± 5.85	1.00 ± 1.55	50.67 ± 5.68	3.17 ± 1.47	52.83 ± 1.60	25.83 ± 5.15	91.50 ± 8.92
6.0	54.50 ± 9.35	144.17 ± 5.04	0.00 ± 0.00	6.83 ± 2.48	1.67 ± 0.52	12.67 ± 1.63	14.33 ± 5.68	45.33 ± 3.78
8.0	52.67 ± 9.54	145.67 ± 2.73	0.00 ± 0.00	0.67 ± 0.82	0.00 ± 0.00	8.00 ± 1.26	0.00 ± 0.00	12.67 ± 1.75
10.0	57.00 ± 8.83	144.50 ± 5.82	0.00 ± 0.00	0.00 ± 0.00	0.00 ± 0.00	12.67 ± 1.97	0.00 ± 0.00	2.67 ± 1.63
25.0	57.67 ± 7.09	143.83 ± 7.19	0.00 ± 0.00	0.00 ± 0.00	0.00 ± 0.00	11.33 ± 0.82	0.00 ± 0.00	0.00 ± 0.00
50.0	49.50 ± 8.07	147.00 ± 3.16	0.00 ± 0.00	0.00 ± 0.00	0.00 ± 0.00	0.00 ± 0.00	0.00 ± 0.00	0.00 ± 0.00
75.0	51.17 ± 6.62	146.67 ± 6.12	0.00 ± 0.00	0.00 ± 0.00	0.00 ± 0.00	0.00 ± 0.00	0.00 ± 0.00	0.00 ± 0.00

The values are the no. of J2s present after the egg hatched. (Data is presented as Mean ± SD of six replicates).

[Where, RE = aqueous root extract of *G. glabra*, GRAgNPs= silver nanoparticles synthesized using aqueous root extract of *G. glabra*, CAgNPs = commercial silver nanoparticles, and VP = commercial nematicide, Velum prime].

Table S3. Effect of RE, GRAgNPs, CAgNPs, and VP on mortality (%) in J2s after 12 h and 24 h of treatment.

Control/ Treatment	RE		GRAgNPs		CAgNPs		VP	
**Concentration (ppm)**	**12 h**	**24 h**	**12 h**	**24 h**	**12 h**	**24 h**	**12 h**	**24 h**
DDW	0.00 ± 0.00	0.00 ± 0.00	0.00 ± 0.00	0.00 ± 0.00	0.00 ± 0.00	0.00 ± 0.00	0.00 ± 0.00	0.00 ± 0.00
0.5	0.00 ± 0.00	0.00 ± 0.00	28.33 ± 2.80	98.83 ± 1.17	0.00 ± 0.00	0.00 ± 0.00	0.00 ± 0.00	0.00 ± 0.00
1.0	0.00 ± 0.00	0.00 ± 0.00	52.50 ± 2.73	99.67 ± 0.52	0.00 ± 0.00	3.17 ± 1.17	0.00 ± 0.00	1.33 ± 0.52
2.0	0.00 ± 0.00	0.00 ± 0.00	86.83 ± 3.81	100.00 ± 0.00	44.83 ± 2.31	57.00 ± 1.41	0.00 ± 0.00	10.83 ± 0.75
4.0	0.00 ± 0.00	0.00 ± 0.00	94.00 ± 2.09	100.00 ± 0.00	74.16 ± 3.06	99.33 ± 0.82	8.50 ± 1.37	24.00 ± 1.26
6.0	0.00 ± 0.00	0.00 ± 0.00	100.00 ± 0.00	100.00 ± 0.00	97.00 ± 0.28	100.00 ± 0.00	31.66 ± 2.58	53.83 ± 2.71
8.0	0.00 ± 0.00	0.00 ± 0.00	100.00 ± 0.00	100.00 ± 0.00	99.83 ± 0.40	100.00 ± 0.00	51.5 ± 2.42	63.83 ± 2.99
10.0	0.00 ± 0.00	0.00 ± 0.00	100.00 ± 0.00	100.00 ± 0.00	100.00 ± 0.00	100.00 ± 0.00	74.66 ± 2.73	76.83 ± 1.94
25.0	0.00 ± 0.00	0.00 ± 0.00	100.00 ± 0.00	100.00 ± 0.00	100.00 ± 0.00	100.00 ± 0.00	85.83 ± 2.85	93.17 ± 2.23
50.0	0.00 ± 0.00	0.00 ± 0.00	100.00 ± 0.00	100.00 ± 0.00	100.00 ± 0.00	100.00 ± 0.00	94.16 ± 1.47	97.33 ± 0.82
75.0	0.00 ± 0.00	0.00 ± 0.00	100.00 ± 0.00	100.00 ± 0.00	100.00 ± 0.00	100.00 ± 0.00	98.33 ± 1.63	99.83 ± 0.41

*Data is presented as mean ± SD of six replicates.

[Where, RE = aqueous root extract of *G. glabra*, GRAgNPs = silver nanoparticles synthesized using aqueous root extract of *G. glabra*, CAgNPs = commercial silver nanoparticles, and VP= commercial nematicide, Velum prime].
